# Commentary: Golgin-97 Targets Ectopically Expressed Inward Rectifying Potassium Channel, Kir2.1, to the *Trans*-Golgi Network in COS-7 Cells

**DOI:** 10.3389/fphys.2018.01401

**Published:** 2018-10-05

**Authors:** Eva-Maria Zangerl-Plessl, Marcel A. G. van der Heyden

**Affiliations:** ^1^Department of Pharmacology and Toxicology, University of Vienna, Vienna, Austria; ^2^Department of Medical Physiology, Division of Heart and Lungs, University Medical Center Utrecht, Utrecht, Netherlands

**Keywords:** K_IR_2.1, TGN, Trafficking, Structure, Golgin-97, drug interference

Taneja et al. (2018) recently reported interesting new findings on anterograde trafficking of the inward rectifying potassium channel K_IR_2.1. By intelligent use of a set of classic and state-of-the-art molecular, cell biological, biochemical, and computational methods they provided compelling evidence for interaction between K_IR_2.1 channel proteins and the Golgi tether protein Golgin-97, and thus identified a mechanism of K_IR_2.1 trafficking through the Golgi system to reach the correct “gate” in trans-Golgi network for its “take-of” to the plasma membrane.

In their work the authors elegantly demonstrate specificity in potassium ion channel trafficking processes in COS-7 cells. K_IR_2.1 proteins use Golgin-97 for their passage through the Golgi system. The Golgin family member GM130, which is implicated in K_v_11.1 (hERG) transport, is however not important for trafficking of either K_IR_2.1 nor K_v_7.1, yet another cardiac potassium ion channel (Roti et al., 2002; Taneja et al., 2018). An earlier example of potassium channel sorting in the Golgi and subsequent trafficking to different subcellular locations by usage of different protein interactions has been revealed for K_v_2.1 and K_v_4.2 in dendrites (Jensen et al., 2014). The indicated complexity of channel specific trafficking processes might be exploited by pharmacological means. For example, the antiprotozoal drug pentamidine decreases K_IR_2.1 and K_v_11.1 expression levels, whereas only the latter could be rescued by dofetilide (Varkevisser et al., 2013). Since K_IR_2.1 channels are widely expressed (de Boer et al., 2010) it raises the question whether the K_IR_2.1-Golgin-97 complex formation shows tissue specificity. Since K_IR_2.1 protein expression and channel function become increased upon atrial fibrillation (e.g., Girmatsion et al., 2009) one can imagine that when Golgin-97 displays sufficient specificity for K_IR_2.1 channels in the atria, a potential drug target might be identified and warrants experimental follow up. Furthermore, short QT syndrome type 3, due to an increase in K_IR_2.1 mediated I_K1_ (e.g., Hattori et al., 2012) may benefit from such a pharmacological approach also when Golgin-97 has a role in ventricular K_IR_2.1 trafficking.

From a structural point of view, it is interesting to point out that the K_IR_2.1 cytoplasmic domain itself can interact with Golgin-97 in the Golgi. It would be interesting to investigate whether this implicates that the cytoplasmic domain (1) binds to the membrane or (2) is transported without direct membrane contact. If the first scenario were to be true, it might give new insights into lipid-protein interaction possibilities, since the cytoplasmic domain does miss key features that usually anchor the protein to the membrane (e.g., the transmembrane domain). It is known from experiments (Hilgemann et al., 2001), that K_IR_2 channels open upon binding of the lipid phosphatidylinositol-4,5-bisphosphate (PIP_2_) to the channel. However, the closely related K_IR_2.2 (Hansen et al., 2011; Lee et al., 2016) crystal structures with PIP_2_ bound are both in the closed state and molecular dynamics simulations do not trigger an opening (Lee et al., 2016). This implicates that there is still a barrier to overcome to get to the open state. If the cytoplasmic domain alone would form lipid interactions, this could be a hint toward full length protein-lipid interactions that are not yet known and that might help the channel to overcome this barrier and induce conformational changes toward its open state.

Based on sequence differences with Golgin-245 in the third helix, the authors chose to test four residues of Golgin-97 for interactions with the cytoplasmic domain. From a structural perspective there would also be other interesting amino acids that might be important for this interaction (e.g., the residue on the helical turn between them). Also interesting is the fact that both mutations, M733K as well as Y740A, lead to a complete lack of channel interaction. This implicates that both residues are major binding determinants. Since they stand in a defined distance to one another, this might help investigating binding of Golgin-97 to the CTD of K_IR_2.1. It would be interesting to check whether the Golgin-97 binding site at the cytoplasmic domain is located at the intracellular lumen accessible surface of the protein or at the interface (N-terminus of the cytoplasmic domain) where there is usually the transmembrane domain. If the interaction site is close to the N-terminus, this might indicate that (1) the binding site might be different for the whole protein, or (2) the final folding of the protein might happen when Golgin-97 leaves the binding site. In most full channel crystal structures of the closely related K_IR_2.2 (Hansen et al., 2011; Lee et al., 2016) and K_IR_3.2 (Whorton and Mackinnon, 2011), the cytoplasmic domain interacts tightly with the slide helix, which is on the N-terminal side of the whole channel. However, there is one crystal structure of K_IR_2.2 (Tao et al., 2009) where PIP_2_ is not bound and the cytoplasmic domain is 6 Ångstrom apart from the (not fully formed) slide helix. It cannot be excluded that this might indicate that only upon release of Golgin-97 the cytoplasmic domain can move toward the slide helix and the channel reaches its completely folded state. As a first step, it would be interesting to see if the crystal structure of the cytoplasmic domain of K_IR_2.1 can form stable interactions with Golgin-97 in a molecular dynamics simulation. If a stable interaction can be found, those results could be tested by mutating the interacting residues on the channel to check if these lead to the same outcome. Therefore, the results obtained in this paper might give first insights and new ideas into the folding process of K_IR_2.1. It would further be interesting to see if these interactions are K_IR_2.1 specific. If they are, this structural information might be a starting point for rationalized drug design to inhibit such interactions (K_IR_2.1-Golgin97) and thus inhibiting forward trafficking thereby reducing functional I_K1_.

## Author contributions

E-MZ-P and MvdH wrote the submitted commentary on an original contribution by Tarvinder Taneja and colleagues.

### Conflict of interest statement

The authors declare that the research was conducted in the absence of any commercial or financial relationships that could be construed as a potential conflict of interest.
